# Therapeut:innen-Augmentation durch generative KI: Konzept und Umsetzung des GAINED-Feedback-Systems an der Universität Osnabrück

**DOI:** 10.1007/s00278-026-00860-2

**Published:** 2026-06-29

**Authors:** Christopher Lalk, Tobias Steinbrenner, Julian Rubel

**Affiliations:** Universität Osnabrück, Lise-Meitner-Str. 3, 49076 Osnabrück, Deutschland

**Keywords:** Feedback in der Psychotherapie, Natural Language Processing, Partizipative Entscheidungsfindung, Qualitätsverbesserung, Ethische Aspekte, Psychotherapy feedback, Natural language processing, Shared decision-making, Quality improvement, Ethical issues

## Abstract

Die zunehmende Verbreitung von generativer KI ermöglicht neue Anwendungsfelder in der Psychotherapie. Dazu zählen 1. Chatbots für eine direkte Interaktion der Patient:innen mit einer KI, 2. Trainingsanwendungen für Therapeuten, bei denen Therapeut:innen mit simulierten KI-Patienten üben können und 3. Augmentationsanwendungen, bei denen Therapeut:innen durch automatisierte Dokumentation oder Feedback- und Entscheidungshilfen unterstützt werden. Gleichzeitig bestehen ethische Risiken durch Datenschutzprobleme, schädlichen Output und Sekundärnutzung klinischer Modelle. In der Psychotherapieambulanz der Universität Osnabrück entwickeln wir unter Berücksichtigung dieser Risiken einen neuen Ansatz zur Therapeut:innen-Augmentation über eine KI-gestützte Feedbackanwendung. Basierend auf robusten inkrementellen Wirkeffekten von Feedback in der Psychotherapie sowie etablierter Forschung zur automatischen Identifikation von therapeutischen Konstrukten in Psychotherapietranskripten erhalten Therapeut:innen Feedback zu Interventionsqualität und Wirkmechanismen. Das Feedback wird mit den Patient:innen besprochen, um eine informierte partizipative Entscheidungsfindung zu fördern. Technische Details sowie ethische und klinische Aspekte der Implementierung werden dargestellt. Abschließend wird ein Ausblick auf zukünftige Entwicklungen und Evaluation der Feedbackanwendung gegeben.

## Abstract (en)

The increasing widespread use of generative artificial intelligence (AI) is opening up new areas of application in psychotherapy. These include 1) chatbots for direct interaction between patients and AI, 2) training applications for therapists, in which therapists can practise with simulated AI patients and 3) augmentation applications, in which therapists are supported by automated documentation or feedback and decision-making tools. At the same time, there are ethical risks due to data protection issues, harmful output and secondary use of clinical models. At the psychotherapy outpatient clinic of the University of Osnabrück we are developing a new approach to therapist augmentation using an AI-supported feedback application, taking these risks into account. Based on robust incremental effects of feedback in psychotherapy as well as established research on the automatic identification of therapeutic constructs in psychotherapy transcripts, therapists receive feedback on intervention quality and mechanisms of action. The feedback is discussed with the patients to promote informed shared decision-making. Technical details as well as ethical and clinical aspects of implementation are presented. Finally, an outlook on future developments and evaluation of the feedback application are provided.

Generative KI eröffnet die Option der substanziellen Erweiterung des psychotherapeutischen Versorgungsangebots. Ein Beispiel stellt das Feedbacksystem „Generative AI for clinical insight and ethical decision-making“ (GAINED) dar. Dieses Verlaufsmonitoring soll als „drittes Ohr“ in der therapeutischen Sitzung fungieren, indem es relevante Inhalte wie Interventionen, Wirkmechanismen und Outcomes systematisch für Patient:innen und Therapeut:innen erfahrbar macht. Die Anwendung soll den wechselseitigen Austausch auf Augenhöhe und die dateninformierte Personalisierung der Behandlung fördern.

## Anwendungsbeispiel aus der Psychotherapieambulanz der Universität Osnabrück

Während die Schnittstelle zwischen Psychotherapie und generativer KI auf der einen Seite neue Anwendungsfelder für Forschung und Praxis ermöglicht, bestehen gleichzeitig aus ethischer Sicht substanzielle Bedenken. Im vorliegenden Beitrag stellen wir unsere Arbeit an der Universität Osnabrück vor, als Beispiel für einen Ansatz, der versucht, beide Seiten ausgewogen zu berücksichtigen. Dabei wird generative KI als Feedbackanwendung genutzt, die Kliniker:innen und Patient:innen ein Verlaufsmonitoring zur Verfügung stellt, das den wechselseitigen Austausch auf Augenhöhe fördern und eine dateninformierte Personalisierung der Behandlung erhöhen soll. Konzeptionell fungiert die Anwendung als eine Art „drittes Ohr“ für die therapeutische Sitzung. Sie macht therapeutisch relevante Inhalte wie Interventionen, Wirkmechanismen und Outcomes systematisch hörbar, ohne die klinische Verantwortung der Therapeut:innen zu ersetzen, und schafft so eine gemeinsame Diskussionsgrundlage für Patient:in und Therapeut:in.

## Natural Language Processing und die Anwendung für Psychotherapieforschung

Auch wenn die KI-basierte Auswertung von Therapieaufzeichnungen in jüngerer Vergangenheit stärker Verbreitung findet, ist die computerbasierte Auswertung von großen Textcorpora schon länger etabliert und wird allgemein als Natural Language Processing (NLP) bezeichnet (Johri et al. 2021). Die Entwicklungen im Bereich NLP erweiterten das Feld der computerisierten Psychotherapieforschung (Imel et al. 2015) durch die Möglichkeit, Therapieoutcomes und Prozesse direkt aus Sitzungsinhalten (z. B. Transkript, akustische Merkmale, Mimik) zu messen, anstatt ausschließlich auf Fragebogen zurückzugreifen. Dadurch können therapeutisch relevante Konstrukte potenziell objektiver und ohne zusätzlichen Erhebungsaufwand erfasst werden.

In Studien unserer Arbeitsgruppe konnte beispielsweise gezeigt werden, dass sprachliche Marker kognitiver Verzerrungen mit einer Depressionssymptomatik assoziiert sind (Lalk et al. 2024b). Weiterhin zeigten sich positive Zusammenhänge zwischen sprachlichen Markern von Vermeidung und der Angstsymptomatik, während Einsichtswörter mit geringerer Symptombelastung einhergingen (Steinbrenner et al. 2025). Durch die Analyse von Sitzungsthemen wurde auch deutlich, dass vor allem gesundheitsbezogene Themen sowie negatives Erleben mit höherer Symptombelastung assoziiert waren (Lalk et al. 2024a). Mit der Entwicklung von generativen Modellen und der Popularisierung von ChatGPT (OpenAI, 2022) ergibt sich schließlich eine Vielzahl weiterer Anwendungen im Bereich der Psychotherapie, wie unten dargestellt.

## Neue Anwendungsfälle der KI im Bereich der Psychotherapie

Generative KI hat in den letzten Jahren substanzielle technologische Sprünge in der Verarbeitung natürlicher Sprache ermöglicht (e.g. Vaswani et al. 2017). Da Psychotherapie eine zutiefst sprachbasierte Praxis ist, ergibt sich daraus ein besonders breites Anwendungspotenzial von neuen Interaktionsformaten zwischen Patient:innen und KI über die Aus- und Weiterbildung von Therapeut:innen bis hin zu klinischer Entscheidungs- und Feedbackunterstützung (Stade et al. 2024). Angesichts der hohen Prävalenz psychischer Störungen mit erheblichem menschlichem Leid und gesundheitssystemischer Belastung (GBD 2019;Mental Disorders Collaborators, 2022) eröffnen diese Entwicklungen zugleich die Aussicht auf eine substanzielle Erweiterung des Versorgungsangebots. Aktuelle Entwicklungen konzentrieren sich auf drei Anwendungsfelder:

1. Therapeutische Chatbots. Sie ermöglichen Patienten eine direkte Interaktion mit KI-Systemen, wobei jedoch ethische Risiken bestehen, wie etwa fehlerhafte Aussagen (Moore et al. 2025) oder die Förderung dysfunktionaler Kognitionen (Steenstra et al. 2026).
2. Trainingsanwendungen für Therapeut:innen. KI-gestützte Chatbots können Patientengespräche simulieren und ermöglichen das Üben therapeutischer Kompetenzen (Zhang et al. 2025). Der Ansatz wird durch Befunde zur *Facilitative Interpersonal Skills Task* gestützt, bei der bessere therapeutische Gesprächsführung mit besseren Therapieergebnissen korreliert (Anderson et al. 2016, 2020).
3. Augmentationsanwendungen für Kliniker:innen. Dies umfasst eine Gruppe von Anwendungen, die Kliniker:innen bei ihrer therapeutischen Tätigkeit entlasten und unterstützen sollen. Darunter fallen zunächst sogenannte *Scribes*, also Tools, die automatisierte Zusammenfassungen oder Dokumentation ermöglichen (Tran et al. 2020). Während diese Anwendungen administrativ entlasten (etwa 10 % bei der Dokumentation; Lukac et al. 2025), umfasst eine andere Subgruppe von Tools klinisches Monitoring und Entscheidungshilfen. Dabei können Therapeut:innen im Therapieverlauf durch Bereitstellung relevanter Informationen oder therapeutischer Interventionen in ihrem Handeln unterstützt werden. Klassisches Routine Outcome Monitoring (ROM), bei dem wiederholt (oft in jeder Sitzung) Fragebogen von den Patient:innen ausgefüllt und die Ergebnisse an die Therapeut:innen zurückgemeldet werden, konnte inkrementelle Verbesserungen des Outcomes, Verringerung der Drop-out-Raten (de Jong et al. 2021) und Verbesserungen der therapeutischen Allianz (Brattland et al. 2019) in zahlreichen Studien und Metaanalysen erzielen. Augmentationen mithilfe von KI könnten die Erweiterung dieser Tools durch automatisierte Erfassung einer großen Bandbreite therapeutisch relevanter Symptome und Wirkmechanismen aus den Therapiesitzungen ermöglichen.

Neben den oben vorgestellten Anwendungsfeldern bestehen zahlreiche weitere Ansätze, wie beispielsweise digitale Navigatoren zur Aufklärung von Patienten bei der Therapieauswahl (Cipriani et al. 2026). Im Folgenden stellen wir mit GAINED (**G**enerative **A**I for clinical **IN**sight and **E**thical **D**ecision-making) einen Augmentationsansatz aus der Psychotherapieambulanz der Universität Osnabrück vor.

## Feedback in der Ambulanz der Universität Osnabrück

Vor dem oben beschriebenen Hintergrund wird im Rahmen des Projekts GAINED eine KI-gestützte Feedbackanwendung in der Psychotherapieambulanz der Universität Osnabrück untersucht. Aufbauend auf den positiven Befunden zu Effekten etablierter Feedbackanwendungen (de Jong et al. 2021) erweitert GAINED die Erfassung von Symptomen um die automatische Einschätzung von therapeutischen Handlungen und Wirkmechanismen auf Basis der Sitzungsaufzeichnung. Dabei werden Wirkmechanismen als Mediatoren des Zusammenhangs zwischen Interventionen und Therapieoutcomes verstanden (Hayes et al. 2022). Dies kann die Personalisierung der Therapie durch ein zusätzliches Monitoring weiterer Konstrukte verbessern, indem z. B. Beziehungsbrüche (z. B. Eubanks et al. 2018) schneller erkannt und adressiert werden können.

Ein User Interface ermöglicht eine visuelle Darstellung des Feedbacks, das gemeinsam mit den Patient:innen besprochen werden kann. Damit sollen Limitationen von herkömmlichen Anwendungen, die Patient:innen oftmals zu wenig einbeziehen, (De Jong et al. 2025), anstatt eine partizipative Entscheidungsfindung (Joosten et al. 2011) zu ermöglichen, adressiert werden. Darüber hinaus soll GAINED die Erhebung therapierelevanter Daten sowie die Auseinandersetzung mit diesen Daten durch die Therapeut:innen erleichtern, welche in der Praxis oftmals am zu hohen Aufwand scheitert (De Jong et al. 2025; Lutz et al. 2021). Durch die Automatisierung der Erhebung, Co-Design mit Psychotherapeut:innen und Patientenvertreter:innen sowie partizipative Diskussion *innerhalb* der Sitzung sollen der inkrementelle Aufwand deutlich reduziert und potenzielle Widerstände gegen die Nutzung abgebaut werden.

## Ethische Aspekte

Das Projekt GAINED wurde nach Ethics-by-Design-Prinzipien konzipiert, d. h., es wurden schon zu Projektbeginn ethische Gesichtspunkte mit Expert:innen, Patientenvertreter:innen und Therapeut:innen diskutiert, um ein umfassendes Konzept zu erarbeiten. Das Konzept wurde im engen Austausch mit einem Lehrstuhl für KI-Ethik erarbeitet. Gleichzeitig fanden zu Projektbeginn bereits Co-Design-Workshops mit Patientenvertreter:innen sowie Therapeut:innen statt. Inhaltlich wurden in den Workshops jeweils erste Ideen präsentiert und diskutiert, wobei der Schwerpunkt auf ethischen Aspekten lag. Dazu wurde auf Patient:innenseite eine Gruppe von Patientenvertreter:innen über die NetzG e. V. (Bundesnetzwerk Selbsthilfe seelische Gesundheit e. V.) rekrutiert und zu einem vergüteten 90-minütigen Workshop eingeladen. Ein zweiter vergüteter Workshop wurde mit dem Patientenvertretung-Board des Treatment and Etiology of Depression in Youth (TEDY) Laboratory am McLean Hospital der Harvard Medical School durchgeführt. Die Patientenvertreter:innen konnten dadurch wichtige Akzente setzen. So wurde beispielsweise darauf hingewiesen, dass KI-Feedback stets als diskussionsanstoßende Perspektive und nicht als autoritative Wahrheit gestaltet sein muss, um kritisches Denken zu fördern und Automatisierungsbias zu vermeiden. Gleichzeitig hoben die Patientenvertreter:innen die Bedeutung von Transparenz und Mitbestimmung hervor, etwa durch nachvollziehbare KI-Aussagen und die Möglichkeit für Patient:innen, aktiv über den Einsatz und die Einsicht in Feedback zu entscheiden. Sie unterstrichen, dass Feedback sensibel und nichtverletzend formuliert sein muss, und betonten die Wichtigkeit der komplementären Erfassung von positiven Konstrukten (z. B. Ressourcen und Stärken, sozialen Beziehungen und positiven Aktivitäten). Auf Therapeut:innenseite wurde ein Workshop mit Therapeut:innen der Psychotherapieambulanz der Universität Osnabrück durchgeführt. Die Rückmeldungen aus den Workshops haben die weiteren ethischen Aspekte und Überlegungen im Wesentlichen geprägt.

Da Psychotherapie in einem geschützten Rahmen stattfindet, in dem der Therapeut einer strengen Schweigepflicht unterliegt, um eine vertrauensvolle und sichere Umgebung schaffen (Oberkircher-Sperling 2014), erfordern die Aspekte von *Geheimhaltung und Datenschutz* besondere Aufmerksamkeit. Die Anwendung generativer KI birgt hier besondere Herausforderungen, da große Sprachmodelle (Large Language Models; LLM) hohe Speicherkapazitäten benötigen, sodass ein Hosting auf einem normalen Anwendercomputer nur unter substanziellen Limitationen (erhöhter Antwortlatenz und stark verminderte Modellqualität) möglich ist. Um ein serverseitiges Hosting und damit einhergehende Risiken für die Geheimhaltung zu umgehen, wird GAINED über einen lokalen Hochleistungsserver gehostet, sodass alle Daten innerhalb der geschützten Ambulanzarchitektur verbleiben. Gleichzeitig findet eine KI-gestützte Weiterverarbeitung der Daten nur bei freiwilliger und informierter Einwilligung der Patient:innen statt, und es besteht die Möglichkeit eines nachträglichen Opt-Out.

Ein weiterer ethisch relevanter Gesichtspunkt ist der *Automation Bias*, also die Tendenz, automatischen, auf Algorithmen basierenden Einschätzungen übermäßiges Gewicht einzuräumen (Goddard et al. 2012). Dieser Aspekt kann insbesondere bei therapeutischen Chatbots in Kombination mit fehlerhaften oder schädlichen Antworten gefährlich sein (Moore et al. 2025): Zum Beispiel erstellten einige LLM im Kontext eines berichteten Jobverlusts auf Nachfrage pflichtschuldig eine Liste von Brücken mit mehr als 25 Metern Höhe in der Umgebung – ohne Bewusstsein für die implizite Suizidalität. Anders als bei klinischen Entscheidungshilfen für Therapeut:innen fehlt hier eine menschliche Kontrollinstanz. Allerdings könnten auch Kliniker:innen bei Entscheidungshilfen oder Feedbacksystemen zu großes Vertrauen in die generierten Daten haben, was im Zweifelsfall insbesondere unter Zeitdruck die klinische Einschätzung verzerren und fehlerhafte Entscheidungen nach sich ziehen könnte (Goddard et al. 2012). Daher sollten explizite Mechanismen implementiert werden, die auf der einen Seite die Zuverlässigkeit des generierten Output erhöhen, während gleichzeitig Nutzer:innen transparent sowohl über die Entscheidungsgrundlage als auch deren Limitationen aufgeklärt werden. Dazu haben wir entsprechende Mechanismen sowohl durch Designelemente (z. B. Anzeige von Konfidenzintervallen, Ausgabe von Erläuterungen des KI-Feedbacks, Hinweise zu fehlerhaften Inhalten) als auch durch Therapeutenschulung (Betonung der Möglichkeit von Halluzinationen, Hinweis auf partizipativen Prozess zur Einordnung des Feedbacks) umgesetzt. In Zukunft soll auch eine Funktion „Feedback the Feedback“ ergänzt werden, womit Therapeut:in-Patient:in-Dyaden direkt zurückmelden können, ob sie dem KI-Feedback zustimmen oder nicht. Dieser zusätzliche Input macht die Anwendung zu einem lernenden System.

Bei unbegleiteten Interaktionen im Rahmen von Chatbots besteht ein zusätzliches Risiko durch übermäßige Nutzung aufgrund der ständigen Verfügbarkeit (Zhang et al. 2025). Zudem fördern die Tendenz von KI-Modellen, Nutzer:innen vorwiegend zuzustimmen (sog. Sycophancy), die Unmittelbarkeit der Antworten und auch proaktive Vorschläge für weitere Auskünfte ganz bewusst die Nutzerinteraktion. Dies kann im schlimmsten Fall Vermeidungstendenzen von Patient:innen verstärken, indem beispielsweise kritische Situationen oder Chatverläufe immer wieder analysiert werden, ohne ins Handeln zu kommen. Damit verbunden ist das Phänomen der *Co-Rumination*, bei dem die Interaktion mit einem Chatbot dysfunktionale kognitive Muster (z. B. Rumination, kognitive Schemata, Größenideen) implizit verstärkt (Steenstra et al. 2026). Um dieses Phänomen zu unterbinden, ist die hier vorgestellte Anwendung explizit für die gemeinsame Nutzung von Patient:in und Therapeut:in vorgesehen und hat keine Chatfunktion. Gleichzeitig fördert dieser Aspekt auf expliziten Wunsch unserer Patientenvertreter:innen sowohl die Transparenz als auch die aktive Beteiligung der Patient:innen an dem Feedbackprozess, sodass für die Patient:innen nachvollziehbar bleibt, welche Informationen genau in dem KI-Feedback dargestellt werden.

Zuletzt ergeben sich aus ethischer Perspektive auch Fragen auf gesamtgesellschaftlicher Ebene. Dies betrifft vor allem das Konstrukt der *Predictive Privac*y (Mühlhoff 2021), also die Möglichkeit, anhand von KI vermeintlich private Informationen durch beiläufig gespeicherte Verhaltensdaten vorherzusagen. So können beispielsweise Social-Media-Konzerne anhand von Nutzerverhalten zuverlässig auf solche privaten Informationen schließen. Im Kontext der Psychotherapie ist dies besonders heikel, da klinische Modelle zur automatisierten Erfassung von Symptomen (z. B. die Erfassung der Depressionssymptomatik anhand von Stimmfeatures) theoretisch auch in anderen Bereichen eingesetzt werden könnten (z. B. im Bewerbungskontext). Diese Sekundärnutzung kann zwar in Teilen bei der Modellentwicklung antizipiert und auch erschwert werden, lässt sich letztlich aber nur auf gesamtgesellschaftlicher Ebene durch Regulierung lösen (Mühlhoff und Ruschemeier 2025). Für das Projekt GAINED bedeutet dies, dass trainierte generative KI-Modelle nicht frei zugänglich sind.

## Technische Aspekte

Zur Infrastruktur der Ambulanz gehören die automatische Aufzeichnung der Sitzungen über Mikrofone sowie die sitzungsweise Fragebogenerhebung von Symptombelastung und Therapieprozessen (z. B. therapeutischer Allianz). Zur Verarbeitung und KI-Inferenz der Daten wird ein interner Hochleistungsserver mit 4 L40S-GPU (je 48 GB GPU-RAM) verwendet. Dies ermöglicht das Hosting von kleinen bis mittelgroßen LLMs (z. B. Mistral-7B, Qwen3-30B) via der Unsloth-Software (Daniel Han 2023). Für die Verarbeitung der Sitzungsaufzeichnungen wird zunächst automatisiert ein qualitativ hochwertiges Transkript erzeugt. Dazu haben wir ein Open-Source-Softwarepaket (Mathew und Lalk 2025) entwickelt, das automatisch Zeitstempel, Sprechertrennung und Transkription durchführt. Unter Training eines LLMs zur Sprecheridentifikation konnten wir die Sprecherzuordnung im Vergleich zur Standardsoftware (75,5 % korrekt zugeordnete Wörter; Taubitz et al. 2025) in einer Teststichprobe von *N* = 22 Therapietranskripten mit 158.586 Wörtern auf 97,1 % verbessern, basierend auf der Word Diarization Error Rate (WDER).

Die relevanten Sitzungsinhalte werden beruhend auf Prosodie und Transkripten automatisiert extrahiert und gemeinsam mit den Fragebogenscores dargestellt. Dies umfasst therapeutisches Handeln (Art und Qualität der angewandten Interventionen), Wirkmechanismen (Mediatoren zwischen Intervention und Outcome) und Outcomes (z. B. Symptomausprägung und Wohlbefinden). In der bisherigen Anwendung werden beispielsweise in Bezug auf therapeutisches Handeln die Ausmaße der herausfordernd-änderungsfokussierten und supportiv-stützenden Interventionen erfasst. Als Wirkmechanismen werden die therapeutische Allianz aus den Fragebogenscores sowie Verhaltensaktivierung und die Umsetzung therapeutischer Aufgaben erfasst. Die Outcomes werden den Fragebogen entnommen und enthalten einen Depressionsscore und einen Score zum Wohlbefinden. Für psychometrisch reliable und valide Messungen durch die KI werden zwei verschiedene Ansätze verfolgt: Zum einen werden LLMs auf Labels von menschlichen Ratern trainiert, unter Verwendung von validierten Labeling-Manualen (*Supervised Learning*). Dabei ermöglichen die Berechnung von Modell-Rater-Übereinstimmung sowie eine Validierung über Fragebogenscores zu verwandten Konstrukten die Sicherung der zentralen psychometrischen Gütekriterien. Alternativ wird unter Verwendung von prompt-basierten Messitems ein Skalenmesswert als Summenscore der Items berechnet (Eberhardt et al. 2025). Einzelne Prompt-Items werden analog zu Fragebogenitems verwendet, um einen latenten Faktor abzubilden. Mittels verschiedenen Prompts werden bei diesem Ansatz also Fragen an ein LLM gestellt, das zusätzlich mit dem Transkript als Kontext „gefüttert“ wird. Ähnlich einer Skala bei einem Fragebogen lassen sich dadurch die modellbasierte Reliabilität und der Modellfit mittels konfirmatorischer Faktoranalyse berechnen, während die Validität anhand von Fragebogen geprüft werden kann.

**Abb. 1:**
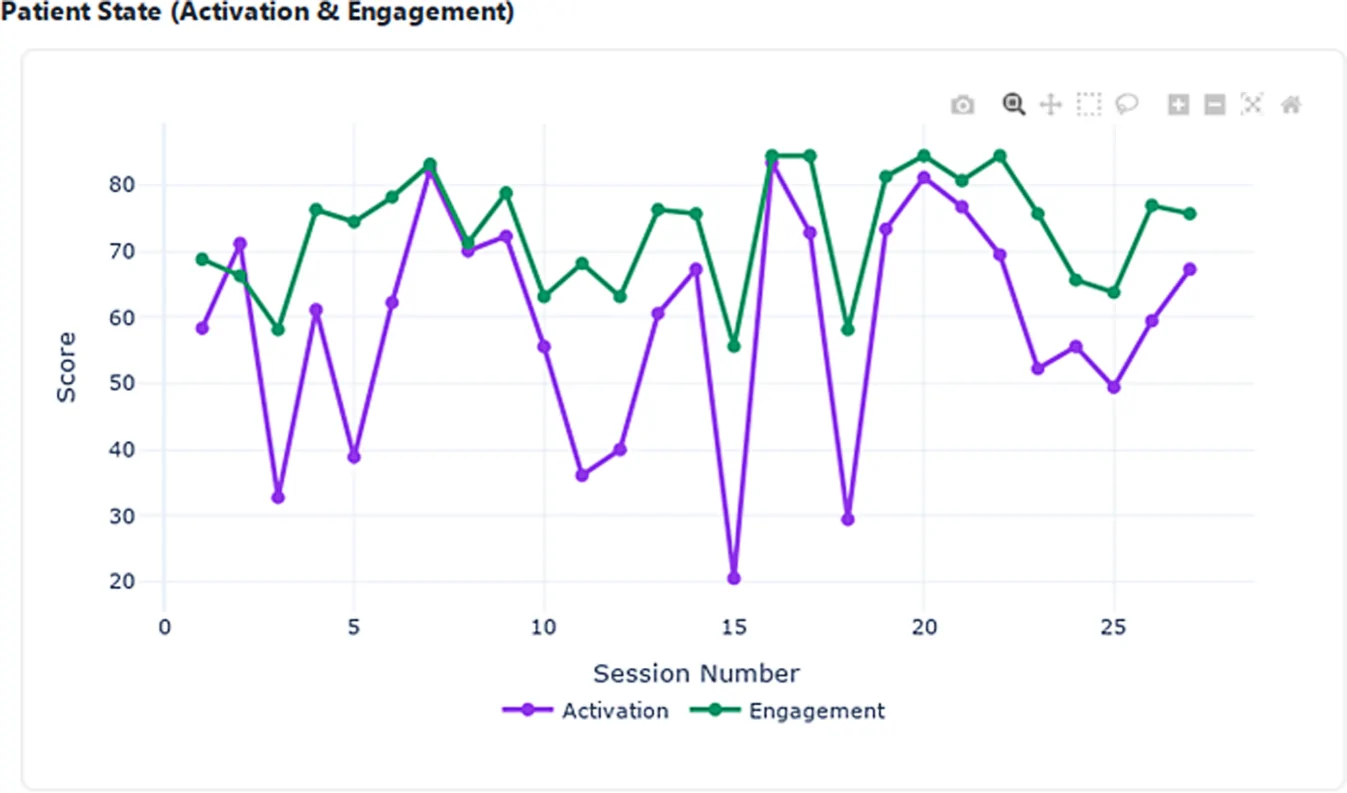
Beispielhafte Darstellung aus dem User Interface. Activation (Aktivitätslevel) und Engagement (Umsetzung therapeutischer Aufgaben) werden über den Therapieverlauf dargestellt. Zusätzlich wird für den jeweiligen Score (hier nicht sichtbar) eine Begründung gegeben

Zur visuellen Darstellung im User Interface werden zunächst einfache Zeitreihen verwendet, die die Ausprägung verschiedener Interventionen, Mechanismen und Outcomes über den Sitzungsverlauf darstellen (Abb. 1). Zur Transparenz und zur besseren Nachvollziehbarkeit der Assessments werden unter Verwendung von strukturierten Outputs einzelne Ratings mit Verweis auf das Transkript erklärt, um Patient:innen und Therapeut:innen eine Einschätzung der Zuverlässigkeit der Messung zu ermöglichen und die KI-Einschätzung gegebenenfalls zu hinterfragen. Für die visuelle Integration der verschiedenen Befunde wird ein Directed Acyclic Graph (DAG) verwendet, der unter Einsatz eines Strukturgleichungsmodells das Zusammenspiel von Interventionen, Mechanismen und Outcomes für den Einzelfall visualisiert.

## Ausblick und offene Fragen

Die vorgestellte KI-Anwendung befindet sich aktuell in der iterativen Entwicklung in der Psychotherapieambulanz der Universität Osnabrück. Dabei ist die Auswertungspipeline von Sitzungsaufzeichnung über die Transkriptgenerierung bis zur Darstellung des KI-Feedbacks über ein User Interface im Rahmen eines voll funktionsfähigen Prototyps umgesetzt. Erste Ergebnisse zu Gütekriterien der KI-Ratings sehen vielversprechend aus, und auch die zeitliche Performance von Transkription, KI-Auswertung und Darstellung ist ausreichend schnell (< 10 min/Einzelsitzung, 5facher Beschleunigungsfaktor). In Zukunft sollen weitere therapeutisch relevante Konstrukte ergänzt werden (z. B. Emotionen, interpersonelle Prozesse), um ein möglichst umfassendes Feedback zu generieren. Darüber hinaus sollen Darstellungselemente noch verständlicher umgesetzt werden, wofür insbesondere weitere Rückmeldungen von Nutzer:innen essentiell sein werden. Aktuell spielen insbesondere das Co-Design und die Pilotierung mit Patientenvertretern:innen, Therapeut:innen und Ambulanz-Patient:innen eine zentrale Rolle. Es soll sichergestellt werden, dass eine erfolgreiche und sichere Implementierung gelingt. Dafür werden erste Rückmeldungen im Rahmen eines Pilot-Tests anhand von qualitativen Interviews mit Patient:innen und Therapeut:innen ausgewertet. Dadurch sollen weitere Anpassungen des User Interface vorgenommen werden, um die Usability sowie die wahrgenommene Nützlichkeit zu optimieren, während gleichzeitig die Risiken reduziert werden. Offene Fragen bestehen aktuell in Bezug darauf, inwieweit es Therapeut:innen gelingt, das Feedback den Patienten erfolgreich zu kommunizieren, und ob dieses als hilfreich erlebt wird. Nach erfolgreicher Entwicklung ist eine Wirksamkeitsprüfung im Rahmen einer randomisierten kontrollierten Studie mit drei Gruppen (kein Feedback, ROM-Feedback, GAINED-Feedback) geplant. Klinische Wirksamkeit durch inkrementelle Effekte ist das zentrale Ziel der Anwendung. Neben der Wirksamkeitsüberprüfung stellen sich insbesondere Fragen zur erfolgreichen Implementierung sowie zu Risiken und negativen Effekten für Patient:innen und Therapeut:innen.

## Fazit für die Praxis

- Bei einer Vielzahl an möglichen Anwendungen gibt es bisher nur wenig Evidenz für inkrementelle Wirksamkeitseffekte von KI in der Psychotherapie.
- Anwendungen der KI in der Psychotherapie gehen mit ethischen und klinischen Risiken einher, die eines intensiven und offenen Diskurses bedürfen. Darunter fallen Aspekte von Geheimhaltung, Automation Bias, exzessiver Nutzung und Co-Rumination sowie einer zweckwidrigen Sekundärnutzung. Therapeutische Chatbots weisen besonders hohe Risiken auf.
- Die erfolgreiche Implementierung ist ein zentrales Problem von therapeutischen Feedbackanwendungen.
- Unser Ansatz zu KI-Feedback soll bisherige Feedbackanwendungen um zusätzliche Informationen, leichtere Implementierung und stärkere Beteiligung der Patienten verbessern – als ein gemeinsam genutztes drittes Ohr für die Sitzung, nicht als autoritative Stimme.

## Data Availability

Es handelt sich um eine Pilotanalyse, in der keine neuen, originären Datensätze generiert wurden. Aufgrund des begrenzten Umfangs wurde kein Daten-Repository erstellt.
